# Patiëntparticipatie bij de ontwikkeling en toepassing van e-health: Bereidheid en voorkeuren van mensen met diabetes mellitus type 2

**DOI:** 10.1007/s12508-021-00313-y

**Published:** 2021-08-03

**Authors:** Catharina M. van Leersum, Marloes Bults, Michelle Sloof, Fabiënne Pouwe, Jeannette G. van Manen, Annemieke A. J. Konijnendijk

**Affiliations:** 1 Universiteit Twente, Enschede, Nederland; 2 Saxion Hogeschool, Enschede, Nederland; 3 Tactus Verslavingszorg, Deventer, Nederland

**Keywords:** patiëntparticipatie, innovatieproces, e‑health, zorginnovatie, diabetes mellitus type 2, Patient participation, Innovation process, eHealth, Health care innovation, Diabetes mellitus type 2

## Abstract

**Inleiding:**

Het doel van dit vragenlijstonderzoek was om in kaart te brengen op welke manieren en op welke momenten mensen met diabetes mellitus type 2 willen participeren bij de ontwikkeling en toepassing van e‑health, en welke factoren daarop van invloed zijn.

**Methode:**

Via verschillende online platforms en de nieuwsbrief van de Diabetesvereniging Nederland is een digitale vragenlijst verspreid met zowel gesloten als open vragen. Informatie werd verzameld over: 1) bereidheid tot participatie; 2) voorkeuren over de vorm van participatie; 3) beïnvloedbare factoren voor participatie, zoals motivatie, competentie, middelen, sociale invloed en uitkomstverwachtingen; 4) achtergrondkenmerken.

**Resultaten:**

Er zijn 160 vragenlijsten geanalyseerd. Ruim 75% van de respondenten heeft interesse in patiëntparticipatie. De meeste respondenten prefereren solistische participatiemethoden boven groepsparticipatie, respectievelijk 93% en 46%. De helft denkt voldoende kennis te hebben om mee te kunnen doen aan patiëntparticipatie en 40% denkt een waardevolle inbreng te kunnen hebben. Als vergoeding wensen deelnemers vooral het gratis gebruik van nieuwe technologie.

**Conclusie:**

Omdat mensen verschillen in hun voorkeuren voor momenten en manieren van participatie, is het aan te bevelen daarvoor verschillende vormen van participatie en vergoedingen aan te bieden tijdens het gehele proces van ontwikkeling tot toepassing van e‑health.

## Abstract (en)

**Aim:**

The aim was to gain insight in the preferences of people with type 2 diabetes mellitus regarding the moments and methods of patient participation in the development and application of eHealth, and which factors influence this.

**Methods:**

A digital questionnaire with both closed and open questions was distributed via various online platforms and the newsletter of the Diabetes Association in the Netherlands. Information was collected on: 1) willingness to participate; 2) preferences about the method of participation; 3) influencing factors on participation, including motivation, competence, resources, social influences, and outcome expectations; 4) background characteristics.

**Results:**

160 questionnaires were analysed. More than three quarter of the respondents intend to be involved in patient participation. Most respondents prefer solo participation methods over group participation, respectively 93% and 46%. Half of the respondents feel that they have sufficient knowledge to participate, and 40% feels that they can provide valuable input. As compensation for participation, participants prefer to use new technologies for free.

**Conclusion:**

As people with diabetes type 2 differ in their preferences for moments and methods of participation, it is recommended to offer different methods of participation and types of compensation in the process from development to application of eHealth.

## Inleiding

Diabetes mellitus type 2 (DMT2) is een van de meest voorkomende chronische aandoeningen in Nederland, met een enorme impact op kwaliteit van leven en zorgkosten [1]. Het aantal mensen met deze vaak leefstijlgerelateerde aandoening neemt snel toe door vergrijzing en ongezonde leefgewoonten [2–4]. Dit zorgelijke vooruitzicht benadrukt het belang van preventie, gezondere leefstijl en zelfmanagement om de prevalentie van DMT2 terug te dringen en om de behandeling van DMT2 te ondersteunen [5]. De komende jaren zal de zorg van een chronisch zieke meer naar de thuissituatie gaan verschuiven, waardoor zelfmanagement, eigen regie en autonomie belangrijker worden [3].

E‑health betreft ‘het gebruik van nieuwe informatie- en communicatietechnologie ter ondersteuning of verbetering van gezondheid en de gezondheidszorg’, zoals gezondheidsapps, platforms of digitale coaches [6]. Dit biedt de mogelijkheid om zorg en begeleiding digitaal en op afstand te organiseren [7]. E‑health heeft grote potentie om mensen met diabetes te ondersteunen bij zelfmanagement in de thuissituatie en om op een andere manier samen te werken met zorgprofessionals. Interventies gericht op aanpassing van leefstijl met ondersteuning van e‑health blijken voor deze patiëntengroep effectief in het verbeteren van de kwaliteit van leven, HbA1c-niveaus, bloeddruk en body mass index (BMI) [8–11].

Desondanks kunnen patiënten verschillende barrières ervaren rond het gebruik van e‑health. Zo kan het zijn dat ze zich niet vertrouwd voelen met e‑health, deze te complex is, niet goed werkt of dat ze geen toegang hebben, of dat de toepassing en het zorgproces niet op elkaar zijn afgestemd [12, 13]. Daarnaast speelt ook het type innovator waarmee iemand zich identificeert een rol bij het accepteren en gebruiken van e‑health. Rogers heeft vijf groepen gedefinieerd die zich onderscheiden in acceptatie van technologie [14]. Deze groepen zijn de innovatoren, pioniers, voorlopers, achterlopers en achterblijvers, waarbij de eerste groep op zoek is naar het nieuwste en de laatste groep liever gebruikt wat ze al hebben [14]. Het type innovator kan een rol spelen bij de bereidheid om te participeren in onderzoek naar e‑health.

Participatie van patiënten bij de ontwikkeling en toepassing van e‑health kan zorgen voor meer aansluiting bij hun wensen, behoeften en mogelijkheden, en hiermee de bereidheid tot het gebruik vergroten [15, 16]. Patiëntparticipatie houdt in dat patiënten de mogelijkheid krijgen om tijdens het innovatieproces hun kennis en ervaringen met hun eigen aandoening in het dagelijks leven in te brengen en zo bij te dragen aan verbeteringen in de zorg [17]. Ervaringen van mensen met een aandoening kunnen erg waardevol zijn, omdat zij op een andere manier naar de zorg kijken dan zorgprofessionals, wetenschappers en ontwikkelaars. Door deze perspectieven te combineren, kan persoonsgerichte zorg ontstaan.

Patiëntparticipatie heeft de afgelopen decennia een enorme ontwikkeling doorgemaakt, waardoor er steeds meer aandacht is voor inbedding van patiëntperspectieven in onderzoek [18]. Patiëntparticipatie kan de rechtvaardigheid van besluiten verhogen, succesvolle implementatie in de zorg faciliteren en de effectiviteit en doelmatigheid van het zorgsysteem vergroten [17, 18]. Daarbij verhoogt patiëntparticipatie de kennis van deelnemers over gezondheid en leefstijl, en activeert ze deelnemers om deze kennis toe te passen bij het bevorderen van gezondheid [19].

Patiëntparticipatie kan op verschillende manieren georganiseerd worden in het gehele proces van e‑healthontwikkeling tot toepassing in de (zorg)praktijk. De CeHRes-routekaart biedt een stappenplan dat helpt om e‑health te creëren waarvoor draagvlak is en die aansluit bij de behoeften van gebruikers [16]. Deze routekaart bestaat uit vijf fases die samen het innovatieproces vormen, namelijk: 1) contextbepaling (in kaart brengen van belanghebbenden, context en zorgsituatie), 2) waardespecificatie (specificeren van waarden en belangen van alle belanghebbenden), 3) ontwerp (ontwerpen van prototype met belanghebbenden), 4) operationalisatie (introduceren en opschalen van de oplossing) en 5) summatieve evaluatie (analyseren van het acceptatiegehalte, gebruik in de praktijk en het behaalde effect) en continue formatieve evaluatie (verzamelen van kennis om vervolgstappen te kunnen maken). In elk van deze fases kan de patiënt een actieve rol spelen.

**Figure 1:**
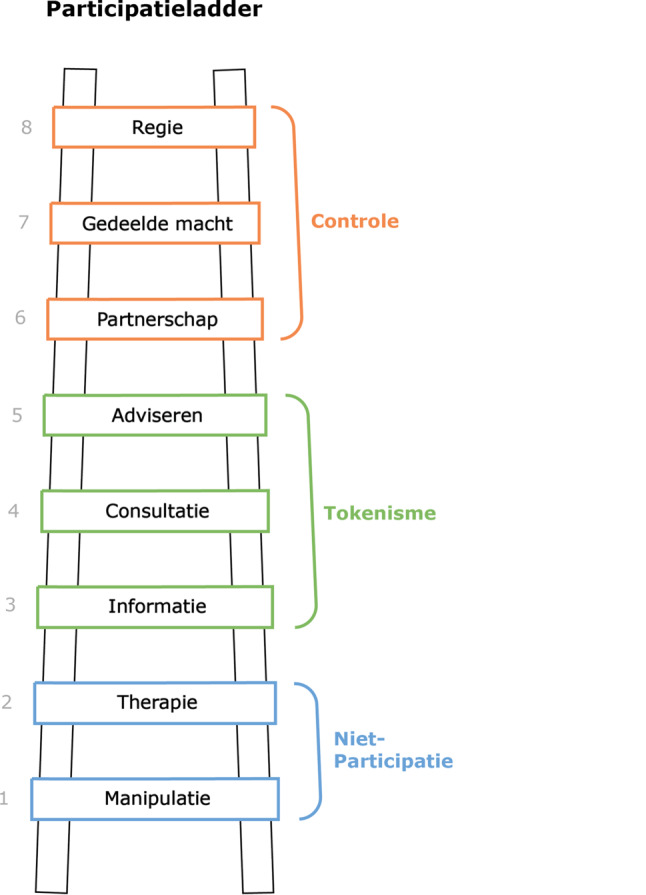

Arnstein definieert de rollen van participanten aan de hand van de participatieladder [20]. Deze acht rollen zijn manipulatie, therapie, informatie, consultatie, adviseren, partnerschap, gedeelde macht en regie. Hiervan worden manipulatie en therapie gezien als niet-participatie. De rollen informatie, consultatie en adviseren worden *tokenisme* genoemd, waarin de burgers een oppervlakkige participatie hebben. Bij de rollen partnerschap, gedeelde macht en regie hebben de participanten steeds meer controle over het onderzoek (fig. 1).

Hoewel de meerwaarde van patiëntparticipatie steeds meer wordt gezien en erkend [17, 18], is er weinig literatuur over de bereidheid van patiënten om te participeren tijdens een e‑healthinnovatieproces. Er zijn evaluatieonderzoeken uitgevoerd om de ervaringen van participanten in beeld te brengen na afloop van een onderzoek [21, 22]. Lemmens et al. identificeerden in een kwalitatief onderzoek meerdere beïnvloedende factoren voor patiëntparticipatie [21]. De beïnvloedende factoren zoals benoemd door de patiënten waren betrokkenheid bij de doelgroep, gevoel van verantwoordelijkheid door eigen ervaring, samenwerking, prioritering van patiëntparticipatie, rekrutering van deelnemers en budget [21]. Het gaat hier echter om ervaringen achteraf en er is geen onderzoek naar de bereidheid en wensen van mensen voorafgaand aan het opzetten van een onderzoek. Er is weinig bekend over de mate en vorm waarin mensen zouden willen participeren bij de ontwikkeling en toepassing van e‑health. Inzicht in de bereidheid, ervaringen en daarop beïnvloedbare factoren is waardevol bij het werven en vormgeven van patiëntparticipatie bij een e‑healthinnovatieproces.

Dit onderzoek is een eerste stap binnen een groter project dat uitgevoerd wordt in het driejarige innovatie- en onderzoeksprogramma TOPFIT Citizenlab (www.topfitcitizenlab.nl). Onderzoekers en Ziekenhuisgroep Twente (ZGT) werken samen aan onder andere participatie van mensen met DMT2 bij het innovatieproces van e‑health. Er is begonnen met mensen met DMT2 omdat er rond deze aandoening veel e‑health ter ondersteuning van leefstijl en zelfmanagement wordt ontwikkeld. Het doel van het project is het ontwikkelen van een persoonsgericht, technologieondersteund aanbod. Dit zou de therapietrouw kunnen verbeteren en kan mensen met DMT2 meer inzicht bieden in de invloed van leefstijl op diabetes. In combinatie met coaching, digitaal of fysiek, geeft dit de mensen met DMT2 mogelijk meer eigen regie, en kan het bovendien de zorgprofessional ondersteunen. Om deze verbeteringen te bewerkstelligen is e‑healthontwikkeling nodig die aansluit bij de behoeften en wensen van mensen met DMT2. Participatie van mensen met DMT2 in deze innovatieprocessen kan zorgen voor de meest passende en bruikbare e‑healthontwikkelingen. Kennis over de participatiewensen kan belangrijke ondersteuning bieden bij het stimuleren van participatie. Het doel van dit eerste onderzoek binnen dit project is om te beschrijven op welke momenten tijdens het innovatieproces en op welke manieren mensen met DMT2 zouden willen participeren.

## Methode

Om de bereidheid en voorkeuren voor participatie van mensen met DMT2 te onderzoeken heeft een cross-sectioneel vragenlijstonderzoek plaatsgevonden. Vanwege de verkennende focus van dit onderzoek is er gekozen voor een vragenlijst om een groep respondenten te bereiken die representatief is voor de populatie mensen met DMT2 in Nederland. Voor de werving van mensen met DMT2 is gebruikgemaakt van verschillende online platforms, zoals LinkedIn, Facebook en Twitter. Daarnaast is er een oproep geplaatst in de online nieuwsbrief van Diabetesvereniging Nederland (DVN) en op het forum van het Diabetes Trefpunt. Inclusiecriteria voor dit onderzoek waren mensen met DMT2 van achttien jaar of ouder die de Nederlandse taal machtig waren. Mensen met ernstige comorbiditeit of psychische stoornissen en/of mensen die geen gebruik kunnen maken van een computer werden geëxcludeerd. Het onderzoek werd bij de ethische toetsingscommissie van de Universiteit Twente goedgekeurd (aanvraagnummer 200589). Respondenten kregen toegang tot de online vragenlijst nadat zij toestemming hadden gegeven via een online toestemmingsformulier.

De vragenlijst bestond uit 33 vragen verdeeld over vier delen:

1. Bereidheid tot participatie op verschillende momenten van een e‑healthinnovatieproces: vier vragen gebaseerd op de CeHRes-routekaart [16], met antwoordopties in de vorm van een vijfpuntslikertschaal (zeker niet tot en met zeker wel). De fases werden benoemd en uitgelegd aan de respondenten: 1) contextbepaling, informatieverzameling over de eindgebruiker, 2) waardespecificatie, informatieverzameling over wat eindgebruikers belangrijk vinden bij een nieuwe technologie, 3) ontwerp, testen van het prototype en 4) operationalisatie en evaluatie, aangepaste technologie inzetten in de praktijk en evalueren van de technologie.
2. Voorkeuren met betrekking tot de vorm van participatie: drie vragen gebaseerd op de participatieladder [20], met antwoordopties in de vorm van een vijfpuntslikertschaal (zeker niet tot en met zeker wel) en drie meerkeuzevragen. Respondenten werd gevraagd of zij individueel of in een groep deel willen nemen aan verscheidene onderzoeksmethoden. De individuele methoden waren een vragenlijst, interviews, het bijhouden van een dagboek of onderdeel zijn van een regelmatig panel. De methoden in een groep betreffen groepsgesprekken met andere mensen met DMT2 of spiegelgesprekken waarbij ook zorgprofessionals aanwezig zijn, deelnemen aan een cliëntenraad, of aan een werkgroep om met de ontwikkelaar of als medeonderzoeker mee te werken in het hele onderzoek.
3. Beïnvloedende factoren voor participatie als motivatie, competentie, middelen, sociale invloed en verwachtingen van innovaties: tien vragen gebaseerd op de beïnvloedende factoren voor participatie [21], met antwoordopties in de vorm van een vijfpuntslikertschaal (zeker niet tot en met zeker wel), drie meerkeuzevragen en twee open vragen naar redenen om wel of niet te participeren in onderzoek.
4. Achtergrondkenmerken van de respondenten: zeven meerkeuzevragen en één open vraag. Hierin is een vraag meegenomen over het type innovator dat past bij de respondent, met daarin innovatoren (‘ik loop meestal voorop in het gebruik van nieuwe technologie’), pioniers (‘ik probeer graag nieuwe technologie voor diabetes uit’), voorlopers (‘ik gebruik technologie pas als ik weet dat het handig en nuttig is’), achterlopers (‘ik gebruik technologie pas als veel mensen die gebruiken’) en achterblijvers (ik gebruik nieuwe technologie niet snel en blijf graag gebruiken wat ik ken’) [14].

Bij de gesloten vragen konden de respondenten antwoordopties selecteren. In de open vragen behorende bij deel 3 werden de respondenten gevraagd om drie redenen te noemen waarom zij wel of niet zouden willen participeren. Hierbij konden de respondenten vrije tekst invullen. Om de validiteit te vergroten werd de vragenlijst ontwikkeld in goed overleg met een groep van zes onderzoekers, twee medewerkers van het ZGT en twee mensen met DMT2.

De vragenlijst werd uitgezet van 4 mei tot en met 10 juli 2020. Het beantwoorden van de vragenlijsten kostte gemiddeld 15 minuten. De gegevensanalyse van de gesloten vragen werd gedaan met SPSS Statistics (versie 24). De analyses betroffen beschrijvende statistiek. Bij iedere vraag werden het aantal respondenten en het percentage dat voor elke antwoordoptie heeft gekozen gerapporteerd. Bij de rapportage van leeftijd en aantal jaren gediagnosticeerd met DMT2 werd een gemiddelde en standaarddeviatie weergegeven.

De gegevensanalyse van de open vragen werd gedaan door twee onderzoekers door middel van een inductieve thematische analyse in Excel. Ieder gegeven antwoord werd gecodeerd en aan ieder antwoord werd een thema gekoppeld. Na het coderen werd een vergelijking van de beschrijvende thema’s gemaakt. Bij elkaar passende thema’s werden gecombineerd, zodat er een codeboom met hoofd- en subthema’s ontstond. Sprekende citaten werden uitgekozen om weer te geven in de resultaten. De resultaten hiervan zijn besproken met meerdere onderzoekers om de betrouwbaarheid en validiteit te verhogen.

## Resultaten

Honderdzestig mensen met DMT2 hebben de vragenlijst volledig ingevuld. De gemiddelde leeftijd was 67 jaar (± 9,6 jaar), de jongste respondent was 27 en de oudste 89 jaar. Gemiddeld hebben de respondenten 16,2 jaar DMT2 (± 8,76 jaar), variërend van 3 maanden tot 47 jaar. Er hebben 13 respondenten met een migratieachtergrond deelgenomen. Daarnaast is gevraagd welk ‘type innovator’ bij de respondenten past (tab. 1).

**Table 1:** 

kenmerk	*n* (%)
*geslacht*	
man	94 (59%)
vrouw	66 (41%)
*leeftijd*	
≤ 40 jaar	2 (1%)
41–50 jaar	8 (5%)
51–60 jaar	24 (15%)
61–70 jaar	63 (39%)
> 70 jaar	63 (39%)
*hoogste afgeronde opleiding*^*a*^	
basisonderwijs	2 (1%)
lbo, vbo, lts, lhno, vmbo (mavo)	29 (18%)
mbo, mts, meao	36 (23%)
havo, vwo, gymnasium	11 (7%)
hbo, heao, pabo, hts	53 (33%)
wo	27 (17%)
*maandinkomen*^*b*^	
minder dan 1000 euro	9 (8%)
1000–2500 euro	61 (53%)
2500–5000 euro	40 (35%)
5000 euro of meer	6 (5%)
*migratieachtergrond*^*a*^	
geen migratieachtergrond	145 (92%)
wel migratieachtergrond	13 (8%)
*duur diabetes*	
≤ 10 jaar	47 (29%)
11–20 jaar	70 (44%)
21–30 jaar	37 (23%)
31-40 jaar	5 (3%)
> 40 jaar	1 (0,6%)
*type innovator* (Rogers innovatietheorie [14])	
loopt meestal voorop in het gebruik van nieuwe technologie	9 (6%)
probeert graag nieuwe technologie voor diabetes uit	69 (43%)
gebruikt technologie pas als respondent weet dat dit handig en nuttig is, en dan ook aanbevelen	58 (36%)
gebruikt technologie pas als veel mensen dit gebruiken en respondent ziet dat dit handig en nuttig is	15 (9%)
gebruikt nieuwe technologie niet snel, respondent blijf graag gebruiken wat hij/zij kent	9 (6%)

^*a*^* N* *=* *158 *vanwege twee ontbrekende antwoorden

^*b*^* N* *=* *116 *vanwege vijf ontbrekende antwoorden en 39 respondenten die hun inkomen liever niet prijsgaven

### Bereidheid tot participatie

Het overgrote deel van de respondenten (79% tot 88%) is bereid of heeft de intentie om actief deel te nemen aan het innovatieproces van e‑health voor mensen met DMT2. Achtentachtig procent van de respondenten wil ‘waarschijnlijk wel’ of ‘zeker wel’ betrokken worden bij het in kaart brengen van het dagelijks leven, de behoeften en de problemen van mensen met DMT2 (de contextbepaling). Daarnaast zou 79% ‘waarschijnlijk wel’ of ’zeker wel’ willen deelnemen aan het in kaart brengen van de wensen en eisen van mensen met DMT2 met betrekking tot een bepaalde technologie (de waardespecificatie). Aan het testen van prototypen, de ontwerpfase, en aan de operationalisatie en evaluatiefase in de praktijk zou 84% ‘waarschijnlijk wel’ of ‘zeker wel’ deelnemen (tab. 2).

**Table 2:** 

fases in het innovatieproces	*n* (%) zeker niet/waarschijnlijk niet	*n* (%) misschien	*n* (%) zeker wel/waarschijnlijk wel
contextbepaling			
*informatieverzameling over de eindgebruikers*			
	6 (4%)	13 (8%)	141 (88%)
waardespecificatie			
*informatieverzameling over wat eindgebruikers belangrijk vinden bij een nieuwe technologie*			
	11 (7%)	23 (14%)	126 (79%)
ontwerp			
*testen van het prototype*			
	9 (6%)	17 (11%)	134 (84%)
operationalisatie en evaluatie			
*aangepaste technologie inzetten in de praktijk en evalueren van de technologie*			
	7 (4%)	19 (12%)	134 (84%)

### Voorkeuren voor de vorm van participatie

Honderdvierenveertig respondenten gaven de voorkeur aan solistische participatiemethoden (tab. 3). Daarvan was de vragenlijst de meest populaire vorm van participatie. Drieënnegentig procent heeft de intentie hier ‘waarschijnlijk wel’ of ‘zeker wel’ aan deel te nemen. Meer dan 50% zou deel willen nemen aan een interview of een panel en 40% zou een dagboek willen bijhouden. Van de 74 respondenten die de voorkeur gaven aan participatie in groepsverband zal een meerderheid ‘waarschijnlijk wel’ of ‘zeker wel’ deel willen nemen aan spiegelgesprekken (77%) of groepsgesprekken (76%). De intentie om onderdeel te worden van een cliëntenraad of een werkgroep werd door meer dan 40% onderstreept en de intentie om medeonderzoeker te zijn werd door 37% onderstreept. Naast de vragen rond een voorkeur voor methoden werden ook praktische zaken uitgevraagd, zoals vergoedingen en tijdinvestering. Wat betreft gewenste vergoeding hebben we hypothetische opties gegeven en daarvan geeft 58% van de respondenten de voorkeur aan gratis gebruik van de technologie die ze hebben getest; 44% geeft reiskostenvergoeding aan als de gewenste vorm van vergoeding. Slechts 19% van de respondenten is bereid om een uur of langer een bijdrage te leveren.

**Table 3:** 

fases in het innovatieproces	*n* (%) zeker niet/waarschijnlijk niet	*n* (%) misschien	*n* (%) zeker wel/waarschijnlijk wel
*solistische vormen van participatie (n* *=* *144)*			
vragenlijst	5 (4%)	5 (4%)	133 (93%)
interview	44 (31%)	20 (14%)	79 (55%)
panel	48 (33%)	20 (14%)	76 (53%)
dagboek	58 (41%)	28 (20%)	57 (40%)
*groepsgewijze vormen van participatie (n* *=* *74)*			
spiegelgesprek	8 (11%)	9 (12%)	57 (77%)
groepsgesprek	9 (12%)	9 (12%)	56 (77%)
cliëntenraad	27 (38%)	13 (18%)	31 (44%)
werkgroep	22 (31%)	20 (28%)	29 (41%)
medeonderzoeker	35 (49%)	10 (14%)	26 (37%)

### Beïnvloedende factoren voor participatie

Tabel 4 geeft weer hoe groot de invloed is van uitkomstverwachtingen, belangrijkheid, sociale omgeving, kennis en zelfeffectiviteit op de bereidheid tot participatie. Naast deze beïnvloedende factoren is het krijgen van een vergoeding voor 18% van de respondenten bepalend.

**Table 4:** 

	beïnvloedende factoren	*n* (%) (helemaal) mee eens
*uitkomstverwachting*	verwachting dat de diabeteszorg verbetert door patiëntparticipatie	140 (88%)
	verwachting dat het eigen leven met diabetes makkelijker wordt door deelname aan patiëntparticipatie	96 (60%)
*belangrijkheid*	belangrijk vinden om mee te praten, zodat het leven met diabetes voor anderen makkelijker wordt	126 (79%)
	belangrijk vinden om mee te praten, zodat eigen leven met diabetes makkelijker wordt	102 (64%)
	belangrijk vinden dat inbreng wordt gebruikt	85 (53%)
*sociale Invloed*	bereidheid tot patiëntparticipatie is hoger wanneer zorgprofessionals dit belangrijk vinden	70 (44%)
	bereidheid tot patiëntparticipatie is hoger wanneer mensen in de omgeving dit belangrijk vinden	46 (29%)
*kennis*	voldoende kennis om deel te nemen aan patiëntparticipatie over nieuwe technologie voor diabetici	78 (49%)
*zelfeffectiviteit*	respondent kan waardevolle inbreng geven over nieuwe technologie	64 (40%)

**Figure 2:**
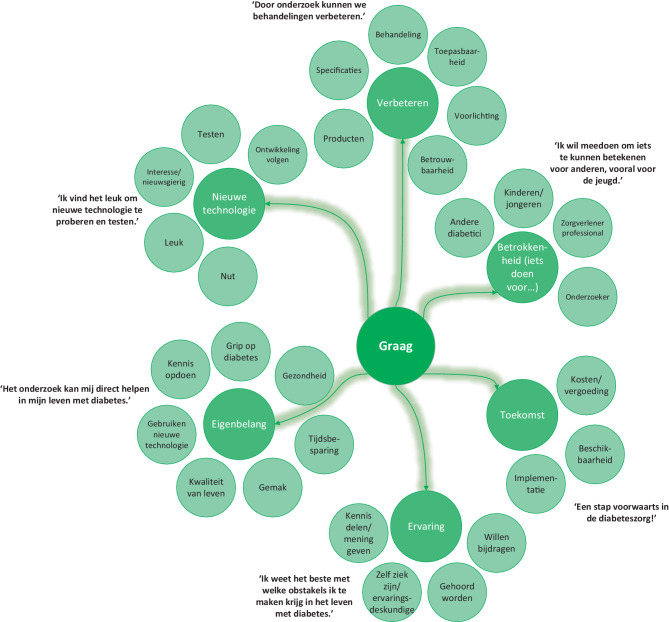

In de open vragen werd een verdiepende slag gemaakt en benoemden de respondenten redenen om wel of niet te participeren in het innovatieproces (fig. 2 en 3). Respondenten gaven aan dat hun ervaring en het kunnen optreden als ervaringsdeskundige een reden is om te participeren: ‘Ik weet wat het is om diabetes te hebben en weet waar ik tegenaan loop in het dagelijkse leven.’ Daarnaast spelen ook redenen als het verbeteren van de technologie of diabeteszorg, en ideeën over de toekomst en implementatie een rol. Respondenten missen bepaalde functies of toepassingsmogelijkheden en zouden hier graag over praten om ‘een stap voorwaarts in diabeteszorg te kunnen maken’. Hierbij vonden de respondenten het idee van misbruik van data of commerciële doeleinden een reden om niet deel te nemen. De technologie moet ook niet te duur worden en mogelijkheden tot vergoedingen vanuit zorgverzekeringen moeten meegenomen worden. ‘Als nieuwe technologie meer macht geeft aan de verzekeraars’, gaven respondenten aan niet te willen participeren.

**Figure 3:**
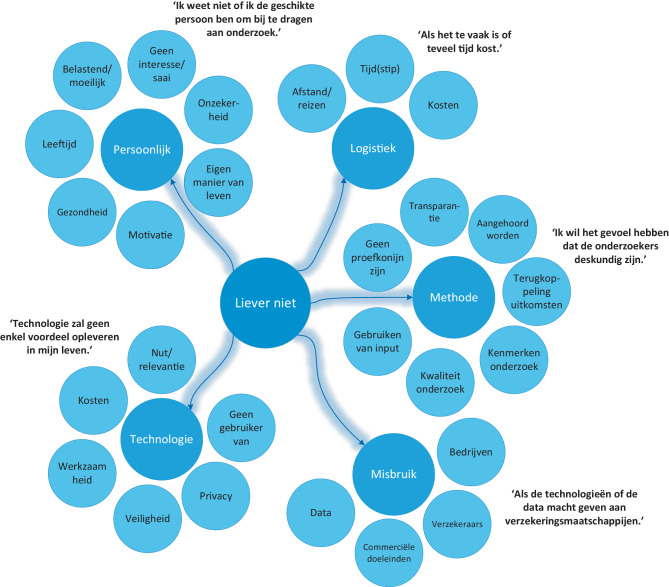

Enerzijds gaven respondenten aan het leuk te vinden, nieuwsgierig te zijn en graag ontwikkelingen op het gebied van technologische innovaties te volgen. Net als in voorgaande vragen werden redenen voor ‘eigen belang’ en ‘belang voor anderen’ veel genoemd. ‘Om te bekijken of de nieuwe technologie iets voor mij of anderen in mijn omgeving is.’ Aan de andere kant gaven respondenten aan technologie ook moeilijk te vinden, kan deze invloed hebben op de privacy en werd niet altijd het nut ervan ingezien: ‘Ik heb geen interesse als de technologie geen noemenswaardig voordeel voor mij oplevert.’ Hiertegenover was het kunnen uitproberen van innovaties een reden om wel te participeren. Meerdere mensen geven aan dat ze graag zelf de technologie thuis testen en toepassen in hun dagelijks leven.

Een aantal respondenten geeft duidelijk aan geen proefkonijn te willen zijn. Vooral participeren in groepsverband wordt benoemd als reden om niet te participeren. Eén respondent benoemde dit als ‘de angst om ondergesneeuwd te worden door verbaal sterke mensen’. Meerdere respondenten geven ook aan dat ze niet deel willen nemen aan interviews als ze ‘twijfel hebben over de deskundigheid van de onderzoeker’ of als ze een andere negatieve invloed ervaren van de onderzoekers of andere respondenten. Aspecten als tijd, reizen en kosten werden ook veelvuldig genoemd als reden om niet te participeren in het innovatieproces: ‘Als het te vaak en te veel tijd in beslag neemt.’

## Beschouwing

### Belangrijkste bevindingen

Tot op heden is er weinig onderzoek gedaan naar momenten tijdens het innovatieproces van e‑health en manieren waarop mensen met DMT2 willen participeren. Uit dit onderzoek blijkt dat de bereidheid onder mensen met DMT2 hoog is om te participeren in iedere fase van het innovatieproces, van contextbepaling tot operationalisatie en evaluatie van e‑health. De voorkeur gaat uit naar participatie via vragenlijsten, groepsgesprekken en spiegelgesprekken. De meeste respondenten willen deelnemen omdat ze verwachten dat patiëntparticipatie de diabeteszorg zal verbeteren of hun leven of dat van anderen met diabetes makkelijker zal maken. Daarnaast wordt inbreng van eigen ervaringen, bijdragen aan verbetering van de technologie en interesse in technologische innovaties genoemd als redenen om te participeren. Het gratis gebruik van technologie die ze kunnen testen wordt gezien als vergoedingsmogelijkheid. Misbruik van data, commerciële doeleinden en kosten werden genoemd als redenen om niet te participeren in het innovatieproces van e‑health.

### Vergelijking met de literatuur

Het nauw betrekken van patiënten bij innovatieprocessen is van belang om de meest passende en gewenste innovaties te realiseren [17, 18]. Er is weinig literatuur over de bereidheid en wensen omtrent de vormen van participatie van mensen voorafgaand aan onderzoek. Een eerder onderzoek naar bereidheid tot participatie laat zien dat mensen willen participeren wanneer het onderzoek gerelateerd is aan een behandeling, maar niet wanneer het risico’s met zich meebrengt of wanneer ze weerstand voelen tegen onderzoek [23]. Een ander onderzoek heeft deelnemers benaderd die niet gereageerd hadden op een uitnodiging tot participatie. Hieruit kwamen vergelijkbare negatieve gevoelens over risico’s en weerstand naar boven, maar ook de verwachte tijdsbelasting werd veel genoemd als reden om niet te participeren [24]. Dit onderzoek laat vergelijkbare resultaten zien met betrekking tot redenen om wel of niet te participeren en voegt daaraan toe op welke manier en in welke vorm mensen zouden willen participeren.

De bereidheid onder mensen met DMT2 om te participeren in iedere fase van het innovatieproces is hoog. Uit dit onderzoek blijkt dat de bereidheid om te participeren in het innovatieproces voor een deel van de respondenten verhoogd kan worden als zorgprofessionals het belangrijk vinden. Daarnaast blijkt ook dat de zelfeffectiviteit van de respondenten laag is. Met andere woorden, ze achten zichzelf niet of nauwelijks in staat om zelf iets in te brengen. Zelfeffectiviteit kan van invloed zijn op de daadwerkelijke participatie [25, 26]. De zorgprofessionals van mensen met DMT2 kunnen een belangrijke schakel vormen. Kennis en overtuigingen van zorgprofessionals vormen een van de vijf categorieën die invloed hebben op de participatie van patiënten [27].

### Sterke en zwakke punten

Aan dit onderzoek deden 160 respondenten mee, voldoende voor een exploratief onderzoek met een deels kwalitatieve focus. Daarnaast laten de achtergrondkenmerken zien dat het om een zeer diverse populatie gaat. Vanwege de diversiteit van de populatie en daarmee lage aantallen van subpopulaties hebben wij geen verbanden gevonden tussen achtergrondkenmerken en de bereidheid tot deelname of wensen voor patiëntparticipatie. Het grotere aantal mannen en de gemiddeld hogere leeftijd van de respondenten is representatief voor de Nederlandse populatie mensen met DMT2. Het aantal respondenten met een migratieachtergrond is lager [26]. Het was wenselijk een hoger aantal respondenten met een migratieachtergrond te bereiken, aangezien DMT2 bij deze populatie relatief veel voorkomt. Met de wervingsmethoden van dit onderzoek werd deze groep niet aangesproken, iets waar vervolgonderzoek naar zal moeten kijken. Daarnaast zal ook rekening moeten worden gehouden met gezondheidsvaardigheden, waarnaar in dit onderzoek geen uitvraag is gedaan. Dit zou een aanbeveling kunnen zijn voor vervolgonderzoek, vooral om te voorkomen dat bepaalde groepen patiënten buitengesloten worden bij onderzoek naar innovaties en dat het gebruik van e‑health daardoor voor hen niet past of mogelijk is.

Uit het innovatieprofiel van de respondenten blijkt dat een groot deel graag nieuwe technologie uitprobeert, wat mogelijk tot selectiebias heeft geleid. In vervolgonderzoek zal naar de representativiteit van deze groep gekeken kunnen worden om eventueel aanvullend onderzoek te doen met bijvoorbeeld mensen met een migratieachtergrond of lage gezondheidsvaardigheden. Daarnaast verwachten we dat een groot aantal van de respondenten al positief is over participeren in onderzoek naar e‑health omdat zij al deelnemen aan een online vragenlijstonderzoek. Vanwege de COVID-19-pandemie hebben we gekozen voor online verspreiding van de vragenlijst, wat mogelijk tot bias in het type respondenten heeft geleid. Bij de verspreiding van de vragenlijst zijn verschillende media gebruikt, waardoor we niet weten welke kenmerken mensen met DMT2 hebben die niet hebben deelgenomen.

Voor het doel van dit onderzoek was geen gevalideerde vragenlijst beschikbaar en daarom is er gebruikgemaakt van een zelfontworpen vragenlijst. De vragenlijst is opgesteld en beoordeeld op begrip door mensen met lage gezondheidsvaardigheden. Daarna is de vragenlijst getest door medewerkers van het ZGT en mensen met DMT2. De huidige wetenschappelijke onderzoeken streven ernaar om er meer respondenten bij te betrekken. Het zou een goede investering zijn om een vragenlijst over wensen voor participatief onderzoek te ontwikkelen en te valideren, zodat onderzoek kan worden gedaan naar de bereidheid en wensen van mensen om te participeren. Verder zou naast kwantitatief onderzoek ook kwalitatief onderzoek moeten worden gedaan. Op die manier kan diepere informatie verkregen worden over manieren en momenten waarop mensen betrokken willen worden. Daarbij kunnen specifieke vragen opgenomen worden om meer te weten te komen over mensen met bijvoorbeeld lage gezondheidsvaardigheden.

### Aanbevelingen voor de praktijk en toekomstig onderzoek

Naar aanleiding van dit onderzoek is een aantal aanbevelingen voor de praktijk en vervolgonderzoek opgesteld. Bij het opzetten van een onderzoeks- en innovatieproject voor e‑health is het van belang de doelgroep in een zo vroeg mogelijk stadium bij het onderzoek te betrekken en gezamenlijk in kaart te brengen welke rol de doelgroep in de verschillende fases van het innovatieproces speelt. Bij het opzetten van een patiëntparticipatieonderzoek moet hier goed over worden nagedacht. Veel respondenten willen bijdragen in de vorm van een vragenlijst, groepsgesprek of spiegelgesprek en er zijn minder respondenten geïnteresseerd in deelname aan panels of andere intensievere vormen. Dit hoeft geen nadeel te zijn, aangezien er voor deze methoden vaak minder mensen nodig zijn. Bij het opzetten van een onderzoek is het aan te bevelen om tijdens de werving ook te kijken naar vormen van participatie. Tijdens de werving is het mogelijk om de deelnemers te laten kiezen tussen gewenste vormen van participatie en mogelijke vergoedingen. Deze keuze maakt patiëntparticipatie mogelijk interessanter voor de deelnemers.

Uit dit onderzoek komt naar voren dat niet alleen financiële vergoedingen tot de compensatiemogelijkheden behoren, maar ook dat het gratis testen en behouden van technologie als een mogelijke compensatie gezien wordt. De duur van participatie heeft ook invloed op de bereidheid: mensen willen er liever niet te veel tijd aan besteden. Het is aan te bevelen om in kleine groepen korte sessies te organiseren, waarbij technologie kan worden uitgeprobeerd en getest. Het is ook aan te raden om zorgprofessionals bij deze sessies te betrekken. Er zou meer aandacht besteed kunnen worden aan de rol van zorgprofessionals door hen nauw te betrekken bij het werven van deelnemers voor het innovatieproces. Daarnaast kunnen zorgprofessionals een sleutelrol spelen door het belang van participatie te benadrukken en de mensen met DMT2 te enthousiasmeren om aan diverse methoden deel te nemen. Op deze manier zou een bredere groep participanten bereikt kunnen worden. In samenwerking zouden bepaalde doelgroepen specifiek benaderd kunnen worden, zoals mensen met een migratieachtergrond of lage gezondheidsvaardigheden.

In dit onderzoek is niet specifiek gevraagd naar de mate waarin met onderzoekers wordt samengewerkt en naar de complexiteit van de wijze van participeren. De samenwerking kan variëren van respondent zijn tot het voeren van de regie. Lemmens et al. laten zien dat mensen die aan onderzoek hebben deelgenomen dat als zeer positief ervaren [21]. Hun vorm van participatie werd in de meeste gevallen informatie of consultatie genoemd op de participatieladder van Arnstein; een enkele keer was er sprake van partnerschap of regie [20]. Onderzoek naar ervaren meerwaarde zoals benoemd door deelnemers aan patiëntparticipatie onderscheidt vijf belangrijke thema’s, namelijk authenticiteit, samenwerking, bevindingen genereren, disseminatie en betrokken willen blijven bij toekomstige onderzoeken [22]. We hebben gevraagd naar de manieren waarop mensen betrokken zouden willen worden. Vervolgonderzoek zou ook specifiek aandacht kunnen besteden aan de wensen met betrekking tot samenwerking met onderzoekers en kunnen kijken naar de ervaringen van de daadwerkelijke patiëntparticipatie. Bij een evaluatie van de ervaringen met patiëntparticipatie onder mensen met DMT2 kunnen de vijf thema’s met betrekking tot ervaring en meerwaarde van patiëntparticipatie gebruikt en mogelijk verder uitgewerkt worden.

## Conclusie

Mensen met DMT2 blijken bereid te zijn om op verschillende momenten en manieren samen te werken in innovatieprocessen. Bij patiëntparticipatie in innovatieprocessen van e‑health kunnen ze samen met hun zorgprofessionals een sleutelrol spelen. Mensen met DMT2 willen op verschillende manieren bij het innovatieproces betrokken zijn. Rekening houdend met alle wensen met betrekking tot patiëntparticipatie kan deze opgezet worden om samenwerking te bevorderen van mensen met DMT2, zorgprofessionals, technologieontwikkelaars, beleidsmakers, andere belanghebbenden en onderzoekers, vanaf de ontwikkeling tot aan de toepassing en succesvolle implementatie van e‑health.
